# Phylogenetic reconstruction of ancestral character states for gene expression and mRNA splicing data

**DOI:** 10.1186/1471-2105-6-127

**Published:** 2005-05-27

**Authors:** Roald Rossnes, Ingvar Eidhammer, David A Liberles

**Affiliations:** 1 Computational Biology Unit, Bergen Centre for Computational Science, University of Bergen, 5020 Bergen, Norway

## Abstract

**Background:**

As genomes evolve after speciation, gene content, coding sequence, gene expression, and splicing all diverge with time from ancestors with close relatives. A minimum evolution general method for continuous character analysis in a phylogenetic perspective is presented that allows for reconstruction of ancestral character states and for measuring along branch evolution.

**Results:**

A software package for reconstruction of continuous character traits, like relative gene expression levels or alternative splice site usage data is presented and is available for download at . This program was applied to a primate gene expression dataset to detect transcription factor binding sites that have undergone substitution, potentially having driven lineage-specific differences in gene expression.

**Conclusion:**

Systematic analysis of lineage-specific evolution is becoming the cornerstone of comparative genomics. New methods, like phyrex, extend the capabilities of comparative genomics by tracing the evolution of additional biomolecular processes.

## Background

Following speciation, there are many possible molecular events that can drive the divergence of species. Three of the most important mechanisms include changes in the coding sequence of proteins that alter protein function, changes in regulatory regions that affect gene expression, and changes in regulatory regions that affect mRNA splicing.

The evolution of protein-coding sequences has been studied systematically in The Adaptive Evolution Database (TAED), where such sequences were grouped into gene families [1]. Within these gene families, the ratio of nonsynonymous to synonymous nucleotide substitution rates (Ka/Ks) was used to detect an excess of nonsynonymous substitution, with positive selection as a proxy for potential functional change. All cases of positive selection were mapped together from the gene tree to the species tree.

No systematic approach has been taken to examine relative gene expression or mRNA splicing in the same way, partly because both appropriate methods and datasets are lacking. One approach to examine the evolution of gene expression is to examine the substitution rate in promoters and look for lineages with excess substitution, analogous to Ka/Ks for protein coding sequences [2]. This can then be correlated with relative expression levels. An alternative approach is to reconstruct ancestral gene expression states and to examine lineages that show a significant change. This has recently been implemented using a maximum likelihood approach for gene expression data [3].

**Figure 1:**
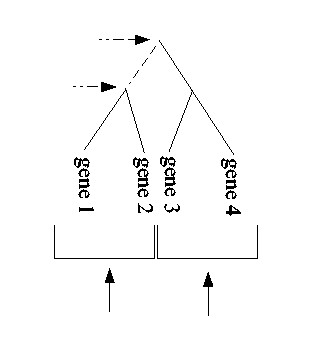
While comparing gene expression values between extant species averages over several branches of a phylogenetic tree, considering change along a branch based upon consideration of ancestral character states increases the signal to noise ratio, resulting in a test with more power to detect causative changes.

Another evolutionary approach to reconstructing ancestral states is minimum evolution. Similar to the principle of parsimony, minimum evolution assumes that significant lineage-specific changes of gene expression through evolution are rarer than conservation of gene expression patterns. Naive comparison of gene expression values of genes across very closely related species does not discourage this assumption. The use of minimum evolution methods to evaluate gene expression ancestral states can be likened to the use of parsimony to evaluate sequence ancestral states and the algorithm is modeled after Fitch parsimony [4]. As shown in Figure 1, examining evolution along branches improves the signal to noise ratio, compared with examining changes between extant sequences resulting in an analysis with more power to detect causative substitutions. As with all methods related to parsimony, the method is expected to be most accurate with short branch lengths and well articulated (speciose) trees.

Using the principle of minimum evolution, a general fast method has been developed that explicitly reconstructs the ancestral state of continuous character traits, like gene expression and mRNA splicing. The speed of this method will enable application to large datasets with many species and readily enables a subsequent mapping of data from gene expression trees to species trees.

Another limitation towards extending TAED-like approaches is the lack of applicable datasets. For mRNA splicing, comparisons of quantitative expressed sequence tag (EST) data and genomic sequence data are used to evaluate relative splicing levels, but existing cross-species comparisons include very long branches [5]. For gene expression, several datasets now exist including closely related species or isolates of yeast [6] and primates [7]. While these datasets are preliminary, they are a starting point to enable testing of methods. Here, we present our minimum evolution method, which is available as free software to download at  and test its performance on the cross-species primate dataset of Enard et al. [7].

## Methods

### Gene Expression and Sequence Data

Gene expression data was collected from Enard et al. [7] and contains samples from brain and liver of human, chimpanzee and orangutan. Sequence data was collected from Ensembl [8] and consists of the sequence 200 bp upstream of the gene transcription start site of the genes in the gene expression dataset.

The reference species tree was taken from Arnason et al., an accepted phylogeny in the field [9].

### Reconstruction of Ancestral Gene Expression States

The reconstruction of continuous characters was done using a minimum evolution approach. A range of values was obtained by running up and down a phylogeny and determining intervals consistent with minimum total evolution over a tree. Once the values converged on final intervals, the mid-point of the range was selected.

**Figure 2:**
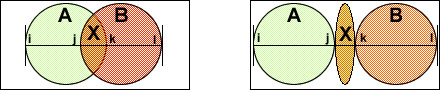
The rules for setting the intervals for ancestral character states are shown diagramatically. If an intersection exists among values at connected nodes, it is the minimum evolution range. If not, the range is the distance between the closest possible values from connected nodes.

**Figure 3:**
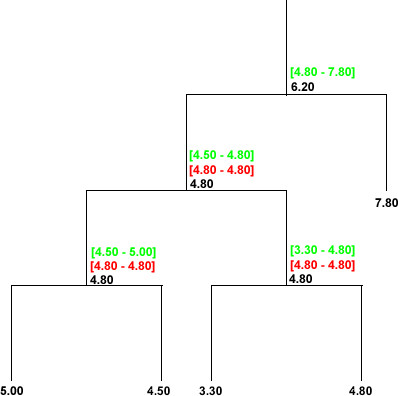
A sample reconstruction of relative gene expression values is shown as the algorithm progresses. Along branch values are then obtained by subtracting the more recent node from the more ancient node. The successive values at each node reflect the values obtained moving up and down the tree as they reach convergence.

**Figure 4:**
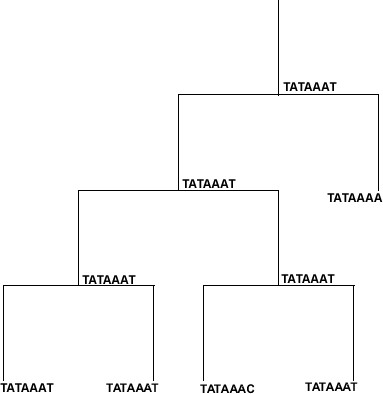
A similar reconstruction of ancestral promoter sequences is shown, using a standard method. Changes along branches in Figure 3 can then be compared with changes along the same branch in Figure 4.

Intervals for the first iteration are taken from those in the descendant leaves or nodes. When they overlap the algorithm calculates the intersection between the intervals, but when they don't the algorithm constructs an interval range between the descendant intervals, as seen in Figure 2. The second iteration ran through the phylogeny to minimize the allowed intervals. This was done by checking the upper and lower limit of the parent node interval and the interval of the node itself. If the upper limit of the parent node was lower than the upper limit of the node itself or the lower limit of the parent node was higher than the lower limit of the node itself, then the limits of the node were changed to be the same as the parent node limits. This is depicted as an example in Figure 3, with a corresponding sequence reconstruction depicted in Figure 4.

### Ancestral sequence reconstruction

ClustalW [10] was used for multiple sequence alignment of promoter sequences and BaseML from the Paml package [11] was used for ancestral sequence reconstruction.

### Along branch analysis

TESS-Transcription Element Search Software [12] was used to search the TRANSFAC database. TESS takes a candidate sequence as input and searches TRANSFAC for transcription factor binding sites that can be locally aligned with regions of the input sequence. The output from TESS consists of a list of transcription factor binding sites that match the input sequence and the position and length of the transcription factor. These lists were manually controlled for each input sequence and correlated with the substitutional information calculated from PAML. Promoters with more than 5% pairwise substitution between human and chimpanzee were discarded. If a substitution occurred within a transcription factor binding site along any branch, it was annotated. Distributions were generated of the amount of along branch change in gene expression and ultimately the number of transcription factor binding sites from TESS that were mutated along any branch. This was normalized by the total amount of substitution to generate an enrichment value.

## Results and Discussion

A software package that utilizes a minimum evolution algorithm to reconstruct ancestral states of continuous character data, like relative gene expression or alternative splicing levels and parse the amount of change to each branch of a phylogenetic tree is presented. This software package is available for download at .

Enard et al. present an analysis of gene expression in a set of genes in brain and liver from human, chimpanzee with orangutan as an outgroup [7]. Using this dataset, we reconstructed ancestral gene expression values at the last common ancestor of human and chimpanzee. The promoter sequences (200 bp upstream of the gene start site) for these genes from human, chimpanzee, and mouse as an outgroup were downloaded from Ensembl [8], aligned, and the last common ancestor sequence from human and chimpanzee was reconstructed using BASEML from the PAML package [11], as described in the methods section.

While enhancers can regulate gene expression over long distances and can be critical to changes in gene expression, many important regulators of transcription are located in the 200 bp immediately upstream of the gene start site [13]. While our knowledge of enhancer function does not permit a fully systematic analysis, analysis of promoter regions can be used to identify a non-exhaustive set of candidates.

**Figure 5:**
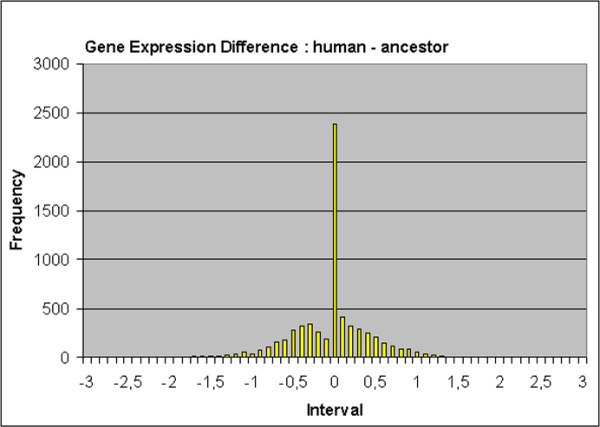
The distribution of gene expression values from Enard et al. [7] obtained with phyrex is shown for the lineage leading from the last common ancestor of human and chimpanzee to human.

**Figure 6:**
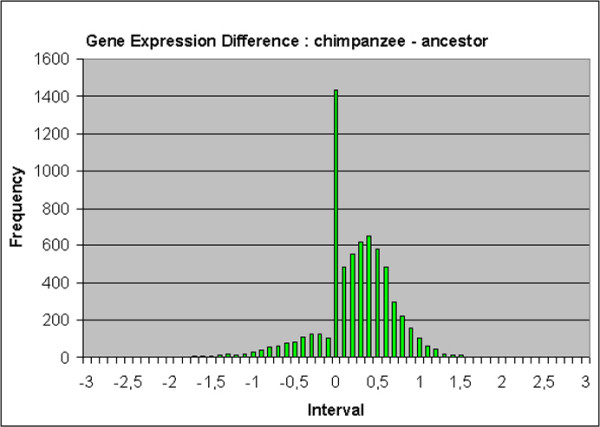
Similarly to Figure 5, the distribution of gene expression values from Enard et al. [7] obtained with phyrex is shown for the lineage leading from the last common ancestor of human and chimpanzee to chimpanzee.

The distribution of gene expression changes across branches is shown in Figures 5 and 6 for the human and chimpanzee lineages, respectively. The strong central peak was expected, given the conservative properties of the method. The asymmetry of the distributions was not expected and may reflect problems with the original dataset. If chimpanzee genes are hybridized to human sequences and then normalized to correct for substitution rates, this type of bias may be expected. However, despite the unexpected shape of the distributions, there is still signal in the data, reflected in the significant enrichment values obtained.

The 0.5% of genes along each lineage that were most upregulated and downregulated were collected with the middle 1% as a control. This corresponded to 34 and 68 genes respectively. Approximately 35% of sequences were eliminated from the analysis because of >5% divergence between human and chimpanzee promoter sequences (the 100 bp more distal to the gene start site were on average much more distant than the 100 bp more proximal to the gene start site and caused most of the elimination). For the remaining sequences, the promoter sequence substitutions that occurred along each lineage were examined for known transcription factor binding sites using TESS [12] and the number of substitutions in such sites evaluated in each group, as shown in Table 1. The total substitution rate was also calculated and used to calculate an enrichment of substitutions in transcription factor binding sites along the lineages with the largest shifts in gene expression. This enrichment, was seen in all four categories, with the human branches showing a better signal to noise ratio than the chimpanzee branches, which may have been expected given the distributions observed in Figures 5 and 6 and the methodology for generating the data.

The supplementary materials  show the actual genes that have been implicated by this analysis, including the prospective transcription factor binding sites that have undergone substitution. The dataset of genes is too small to pick out significant gene function signal from the upregulated and downregulated genes along each lineage. Along the human lineage, there were fewer substitutions predicted to destroy transcription factor binding sites on the up-regulated gene lineages compared with the control, while other lineage data were not different from the control. Because it is not clear which destroyed binding sites are normally occupied by transcriptional activators, it is difficult to interpret the biological significance of this result. While the binding sites predicted may be candidates for playing an important role in the lineage-specific divergence of human and chimpanzee and warrant further testing for their activity in regulating expression from the respective promoters, little experimental data is currently available to further validate the study beyond the statistical validation seen in the enrichment values. However, evolutionary approaches that consider along branch change as opposed to pairwise comparison of extant sequences (as in Figure 1) do hold promise in pinpointing substitutions that cause the divergence of gene expression during species diversification.

## Conclusion

All together, a method (and software) are made available for analysis of gene expression and alternative splicing shifts in a phylogenetic context and for detecting substitutions responsible for driving such shifts. Given some of the approximations made (enhancers ignored, minimum evolution rather than maximum likelihood, asymmetrical dataset to start with), the method performs surprisingly well and is a valuable starting point for this type of analysis, as well as being subject to future improvements. Ultimately, it will be valuable in comparative genomics to compare lineage-specific changes in gene content and in coding sequences, with changes in gene expression and alternative splicing to get a fuller picture of evolution.

## Authors' contributions

This project was conceived by DAL. All programming and analysis was performed by RR, under the supervision and with the technical support of DAL and IE. The paper was written by DAL.

## Availability and requirements

Project name: Phyrex

Project home page: 

Operating systems: Linux

Programming language: Java

Other requirements: Java 1.4.2

License: none

Any restrictions to use by non-academics: none

## Figures and Tables

**Table 1:** The average number of substitutions that occurred in transcription factor binding sites in genes at the tails and center of the distributions are shown. When normalized by the total substitution rate in these promoters, the enrichment of transcription factor binding site substitution detection is shown.

	***Downregulated***	***Unchanged***	***Upregulated***
Human	0.64 ± 0.25 enrichment: 1.48x	0.42 ± 0.01	0.92 ± 0.40 enrichment: 1.71x
Chimpanzee	0.48 ± 0.24 enrichment: 1.39x	0.59 ± 0.15	0.90 ± 0.28 enrichment: 1.36x
